# Blockage of O-linked GlcNAcylation induces AMPK-dependent autophagy in bladder cancer cells

**DOI:** 10.1186/s11658-020-00208-x

**Published:** 2020-03-10

**Authors:** Lu Jin, Feng Yuan, Guangcheng Dai, Qiu Yao, Han Xiang, Lixia Wang, Boxin Xue, Yuxi Shan, Xiaolong Liu

**Affiliations:** Department of Urology, The Second Affiliated Hospital of Soochow University, 1055 Sanxiang Road, Suzhou, 215004, R.P China

**Keywords:** O-GlcNAcylation, Autophagy, ULK1, AMPK

## Abstract

**Background:**

High levels of the post-translational modification O-GlcNAcylation (O-GlcNAc) are found in multiple cancers, including bladder cancer. Autophagy, which can be induced by stress from post-translational modifications, plays a critical role in maintaining cellular homeostasis and regulating tumorigenesis. The impact of O-GlcNAcylation on autophagy in bladder cancer remains unclear. Here, we evaluate the change in autophagic activity in response to O-GlcNAcylation and explore the potential mechanisms.

**Methods:**

O-GlcNAcylation levels in bladder cancer cells were altered through pharmacological or genetic manipulations: treating with 6-diazo-5-oxo-norleucine (DON) or thiamet-G (TG) or up- and downregulation of O-GlcNAc transferase (OGT) or O-GlcNAcase (OGA). Autophagy was determined using fluorescence microscopy and western blotting. Co-immunoprecipitation (Co-IP) assays were performed to evaluate whether the autophagy regulator AMP-activated protein kinase (AMPK) was O-GlcNAc modified.

**Results:**

Cellular autophagic flux was strikingly enhanced as a result of O-GlcNAcylation suppression, whereas it decreased at high O-GlcNAcylation levels. Phosphorylation of AMPK increased after the suppression of O-GlcNAcylation. We found that O-GlcNAcylation of AMPK suppressed the activity of this regulator, thereby inhibiting ULK1 activity and autophagy.

**Conclusion:**

We characterized a new function of O-GlcNAcylation in the suppression of autophagy via regulation of AMPK.

**Graphical abstract:**

Blockage of O-linked GlcNAcylation induces AMPK dependent autophagy in bladder cancer cells. 
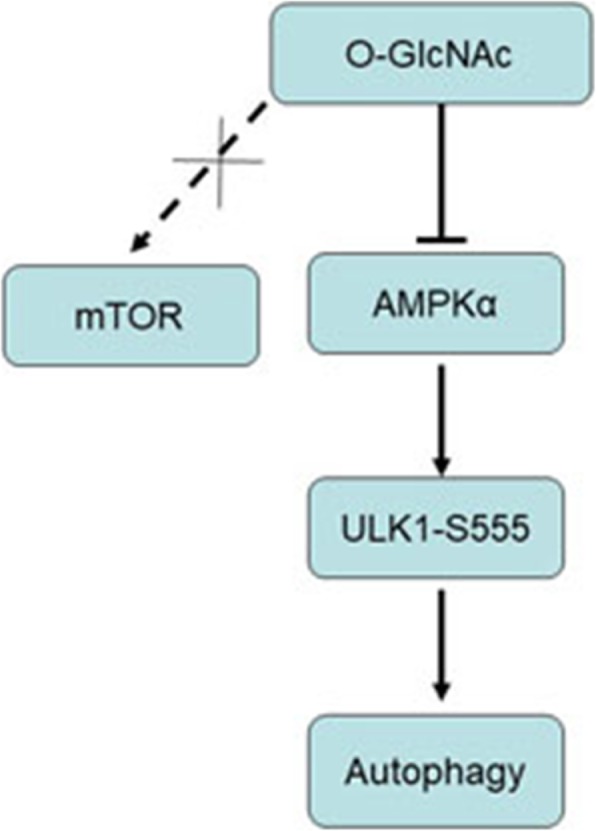

## Background

Bladder cancer is the second most common genitourinary malignancy, accounting for approximately 20% of the cases and mortality in this class worldwide [1]. As with most cancers, its cells have a large demand for nutrients from their environment, leading to an altered metabolic state [2, 3].

The hexosamine biosynthetic pathway (HBP) lies at the nexus of cellular metabolism, utilizing metabolites produced in various anabolic signaling pathways to generate the nucleotide sugar uridine diphosphate N-acetylglucosamine (UDP-GlcNAc). UDP-GlcNAc is the donor sugar for protein glycosylations, including the post-translational modification of nuclear and cytoplasmic proteins with O-linked-β-N-acetylglucosamine, a process mediated by the enzyme O-GlcNAc transferase (OGT) [4, 5]. O-linked-β-N-acetylglucosamine is removed from O-GlcNAc-modified proteins by the glycoside hydrolase O-GlcNAcase (OGA) [6]. O-GlcNAcylation can regulate protein functions by competing with phosphorylation on the same or proximal sites in proteins [7].

Similar to other post-translational modifications, O-GlcNAcylation plays important roles in the regulation of multiple physiological and pathophysiological processes, such as cell signal transduction, transcription, cell division, metabolism and cytoskeletal maintenance. Studies have found that increased levels of O-GlcNAcylation or OGT are involved in the genesis and development of various tumors, including bladder cancer [8–10]. Tumor suppressors and oncoproteins, such as p53, MYC, NF-κB and β-catenin, are modified by O-GlcNAcylation [11–15].

It is well known that an increase in cancer risk is associated with aging, and that aging-related metabolic changes act as drivers of tumorigenesis [16]. Autophagy exerts anti-aging effects in proliferative and post-mitotic cells [17]. As a response to various stresses, including nutrient, oxygen and growth factor deprivation and chemotherapeutics [18, 19], autophagy plays an important role in maintaining cellular homeostasis and regulating tumorigenesis and progression.

It has been confirmed that autophagy contributes to tumor suppression through autophagic removal of potential oncoprotein p62/SQTM1 [20]. Under metabolic stress, AMP-activated protein kinase (AMPK) is activated, triggering autophagy mainly through inhibition of the anti-autophagic mTOR pathway and direct phosphorylation of ULK1 (also called autophagy-related gene 1, ATG1) [21, 22]. Activated ULK1 phosphorylates and activates various autophagy mediators, such as ATG9 and beclin, which are involved in autophagic initiation and progression [5]. In breast cancer cells, Ferrer et al. found that silencing OGT blocks the mTOR pathway and increases the activation of LKB1/AMPK signaling [23].

Based on these findings, we speculate that O-GlcNAcylation may be associated with AMPK-induced autophagy in bladder cancer cells. Here, we demonstrate that O-GlcNAcylation of AMPK suppresses its activity, thereby inhibiting ULK1 activity and autophagy. Our findings might have important implications for the role of O-GlcNAcylation in cancer initiation and progression through disruption of autophagy.

## Methods

### Antibodies, chemicals, and plasmids

Antibodies against p62 (#610832) were purchased from BD Pharmingen. Antibodies against GAPDH (#5174), LKB1 (#3050S), mTOR (#2972), p-mTOR (S2448; #2971S), AMPKα (#2603), p-AMPK (T172; #2535), ACC (#3676), p-ACC (S79; #3661), ULK1 (#8054) and p-ULK1 (S555; #5869) were purchased from Cell Signaling. Polyclonal antibodies against OGT (#SAB2101676) and OGA (#SAB4200311) were purchased from Sigma-Aldrich. The monoclonal antibody against O-GlcNAc (RL2; #MA1072) was purchased from Thermo Fisher Scientific. Antibodies against LC3 (#NB100-2220SS) were purchased from Novus Biological. The anti-GFP monoclonal antibody (#SC9996) was purchased from Santa Cruz Biotechnology. DON, TG and bafilomycin A1 (Baf A1) were purchased from Sigma-Aldrich. The pcDNA6.2-myc construct containing AMPKα, OGT, OGA or null control and the pLKO shRNA construct containing OGT, OGA, AMPKα or negative control were purchased from Addgene.

### Cell lines and gene transfection

The 5637 and RT4 cells were grown in Dulbecco’s modified Eagle’s medium (DMEM) containing 10% fetal bovine serum (FBS) in a humidified incubator containing 5% CO_2_ at 37 °C. The 5637 and RT4 cells were transfected with GFP-LC3, and then positive, stable clones were selected by growing the cells with G418 (800 μg/ml) for 2 weeks. The pcDNA6.2-myc construct containing AMPKα, OGT, OGA or null control was transiently transfected into the 5637-GFP-LC3 cells. All transfections were performed with FuGene 6 transfection reagent (Roche Diagnostics).

### Establishment of stable cell lines

Constructs for shRNA-Ctrl, shRNA-OGT and shRNA-OGA were purchased from Addgene and packaged with the lentiviral expression system. The 5637 and RT4 cells were infected with lentiviruses expressing shRNAs and selected as previously described [24].

### Fluorescence microscopy

The location and distribution of GFP-LC3 staining were examined directly as described previously using a Nikon Eclipse TE2000-E fluorescence microscope [25]. GFP-LC3 puncta were counted manually with the Adobe Photoshop counting tool. GFP-LC3 puncta in three independent assays were calculated by three researchers blindly and the average number ± SD (standard deviation) was shown.

### Western blotting and co-immunoprecipitation (co-IP)

Cells were collected in RIPA lysis buffer. Western blotting was performed as described previously [25]. A total of 30 μg proteins were used for the western blotting unless otherwise indicated. GAPDH was used as the internal control. For Co-IP, total cell lysates were prepared using IP buffer consisting of 20 mM HEPES (pH 7.9), 1 mM EDTA, 1 mM EGTA, 150 mM NaCl and 0.5% IGEPALCA-630. After preclearing with protein A beads, lysates were incubated with antibodies or control IgG overnight at 4 °C. The immune complexes were incubated with protein A beads for 1 h and washed with the same buffer six times. The samples were eluted using the sample buffer, followed by SDS-PAGE and western blotting. All western blots were performed 3 times and quantified once using NIH ImageJ. Expression values were calculated relative to GAPDH.

### Statistical analysis

Student’s *t*-test was used for statistical analyses between two groups. Data are presented as the means ± standard deviations (SD). All statistical analyses were performed using SPSS statistical software version 18.0 and GraphPad Prism 7 software. *p* < 0.05 was considered statistically significant.

## Results

### O-GlcNAcylation negatively regulates autophagy in bladder cancer cells

To determine whether autophagy is regulated by O-GlcNAcylation at basal levels in bladder cancer cell lines, we examined the autophagic flux in cells treated with DON or TG. As an inhibitor for different glutamine utilizing enzymes, DON inhibits the formation of UDP-GlcNAc, thereby reducing O-GlcNAcylation [26, 27]. TG inhibits OGA, thereby promoting O-GlcNAcylation.

**Fig. 1:**
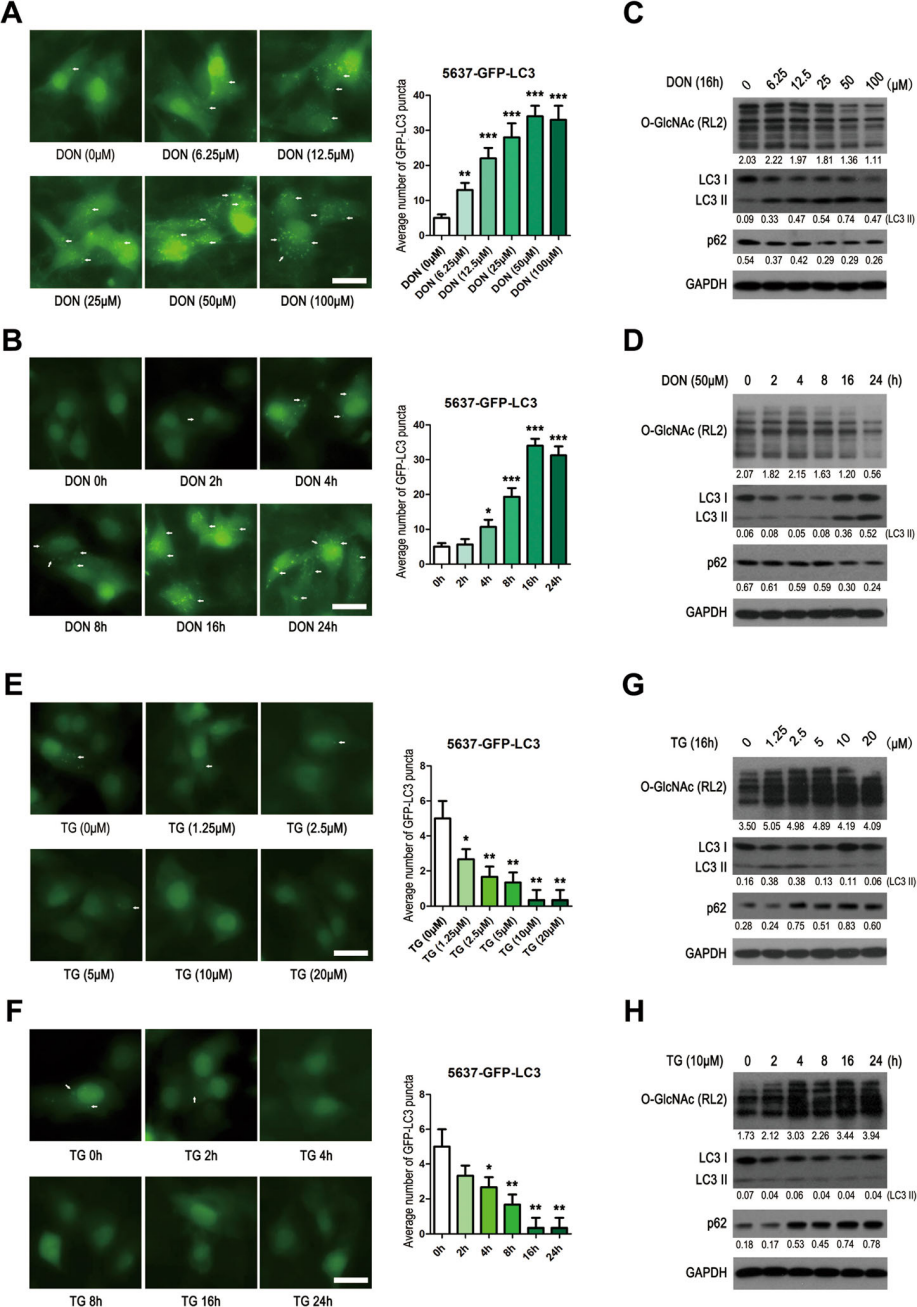
O-GlcNAc negatively regulates autophagic flux. **a –** The 5637-GFP-LC3 cells were treated with different doses of DON. GFP-LC3 fluorescence was captured with fluorescence microscopy 16 h later. **b** – The 5637-GFP-LC3 cells were treated with DON (50 μM). GFP-LC3 fluorescence was captured with fluorescence microscopy at different time points. **c** and **d** – The expressions of O-GlcNAc (RL2), LC3 I, LC3 II and p62 in 5637 cells described in (**a**) and (**b**) were determined using western blotting assays. **e** – The 5637-GFP-LC3 cells were treated with different doses of TG. GFP-LC3 fluorescence was captured with fluorescence microscopy 16 h later. **f** – The 5637-GFP-LC3 cells were treated with TG (10 μM). GFP-LC3 fluorescence was captured with fluorescence microscopy at different time points. **g** and **h** – The expressions of O-GlcNAc (RL2), LC3 I, LC3 II and p62 in the cells described in (**e**) and (**f**) were determined using western blotting assays. The average number of GFP-LC3 puncta was calculated in 200 cells. Data in the histograms are shown as the means ± SD. Scale bar: 20 μm. *p < 0.05, **p < 0.01, ***p < 0.001

To assess autophagy, we used two bladder cancer cell lines, 5637 and RT4, which were stably transfected with GFP-LC3. The number of cytoplasmic puncta of GFP-LC3 was gradually increased in 5637 cells treated with DON at different doses or over time gradients compared with the basal cells (Fig. 1a and b). The western blotting results show a general trend of decreased O-GlcNAcylation and expression of p62, and increased expression of LC3 II (Fig. 1c and d). O-GlcNAcylation and p62 levels were clearly reduced and the LC3 II level clearly increased at the higher dose (50 and 100 μm; Fig. 1c) and the later time (16 and 24 h; Fig. 1d). The p62-degradation indicates an enhanced autophagic flux [20].

By contrast, decreased numbers of GFP-LC3 fluorescent vesicle puncta were observed in 5637 cells treated with TG at different doses (Fig. 1e) or for various times (Fig. 1f). In addition, TG treatment led to an increase in O-GlcNAcylation and p62 levels and a reduction in LC3 II expression (Fig. 1g and h). Similarly, increased numbers of GFP-LC3 cytoplasmic puncta were observed in RT4 cells treated with DON. The numbers of GFP-LC3 cytoplasmic puncta decreased in cells treated with TG compared with cells treated with Mock, although the difference is not significant. (Supplementary Fig. S1A). These results suggest that the O-GlcNAcylation level is negatively related to autophagy in bladder cancer cells.

**Fig. 2:**
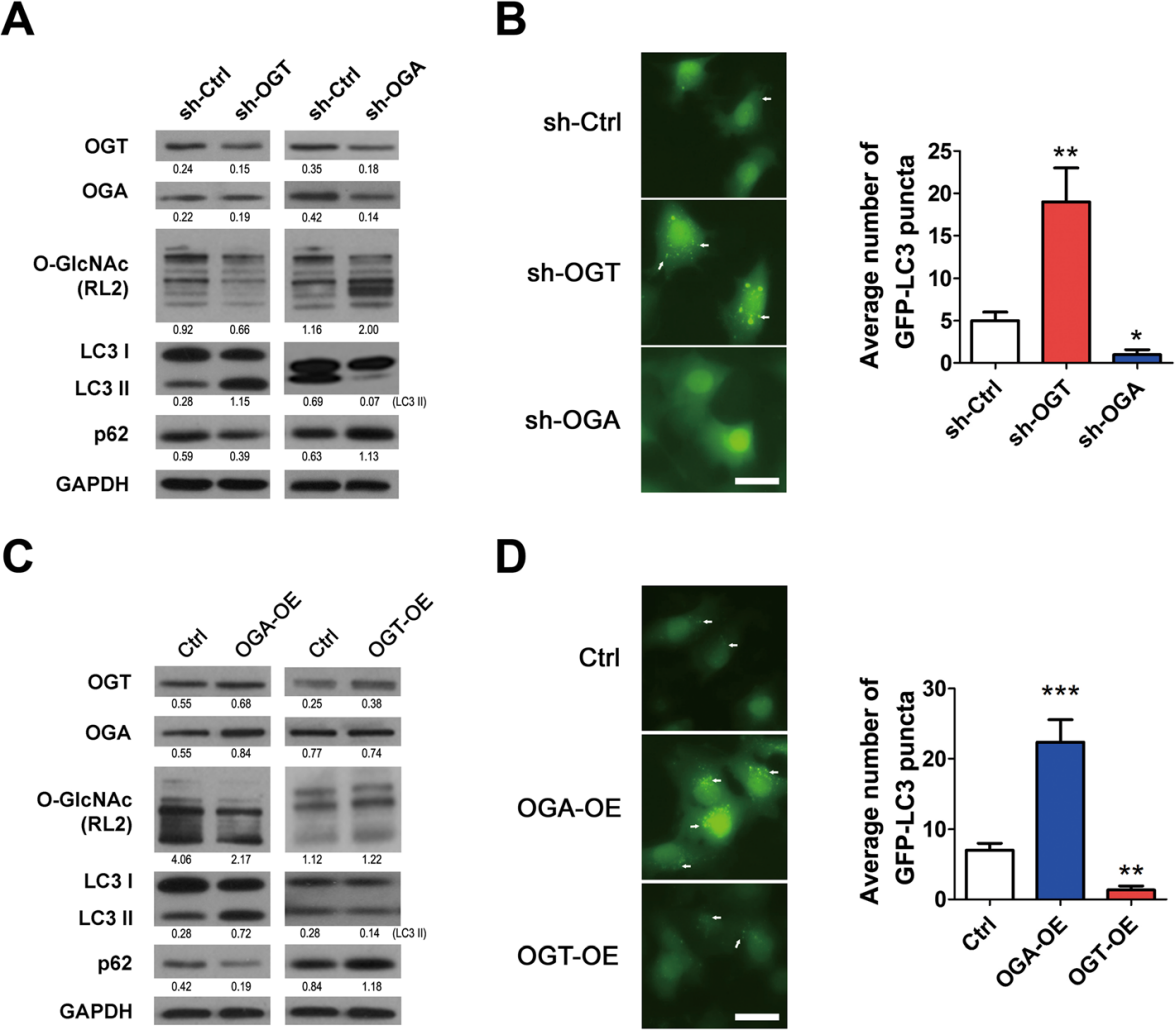
Manipulations of OGT or OGA alter autophagic flux. **a** – Expressions of OGT, OGA, O-GlcNAc (RL2), LC3 I, LC3 II and p62 in 5637-GFP-LC3 cells with OGT or OGA knockdown were determined using western blotting. **b** – GFP-LC3 fluorescence was captured with fluorescence microscopy in the stable cells described in (**a**). **c** – The expression levels of OGT, OGA, O-GlcNAc (RL2), LC3 I, LC3 II and p62 in 5637-GFP-LC3 cells with OGT or OGA overexpression (OE) were determined using western blotting. **d** – GFP-LC3 fluorescence for the cells described in (**c**) was captured with fluorescence microscopy. The average number of GFP-LC3 puncta was calculated for 200 cells. Data in the histograms are shown as the means ± SD. Scale bar: 20 μm. *p < 0.05, **p < 0.01, ***p < 0.001

To further validate the contribution of O-GlcNAcylation to autophagy at the basal level, we changed the global O-GlcNAcylation levels by knocking down the expression of OGT or OGA using specific shRNAs. Knockdown of OGA also inhibited OGT, which might be compensatory regulation of cells. Decreased global levels of O-GlcNAcylation, upregulated LC3 II expression and downregulated p62 expression were detected in sh-OGT stable 5637 cells compared with the control cells (sh-Ctrl) based on the western blotting results (Fig. 2a). By contrast, knockdown of OGA (sh-OGA) in 5637 cells increased the global levels of O-GlcNAcylation and p62 expression and inhibited LC3II expression when compared with sh-Ctrl cells. The expression of LC3 II and p62 altered by OGT or OGA silencing were mirrored by changes in the number of GFP-LC3 cytoplasmic puncta (Fig. 2b).

On the other hand, overexpression of OGA significantly reduced the levels of O-GlcNAcylation and p62, increased LC3 II expression, and promoted the induction of autophagosomes (Fig. 2c and d). Overexpression of OGT had the opposite effect on these markers (Fig. 2c and d). Additionally, increased or decreased numbers of GFP-LC3 cytoplasmic puncta were observed in RT4-GFP-LC3 cells in which OGT or OGA was silenced (Supplementary Fig. S1B). These results support our notion that O-GlcNAcylation contributes to the regulation of autophagy in bladder cancer cells under fully-fed conditions.

### Blockage of O-GlcNAcylation enhances autophagy flux

**Fig. 3:**
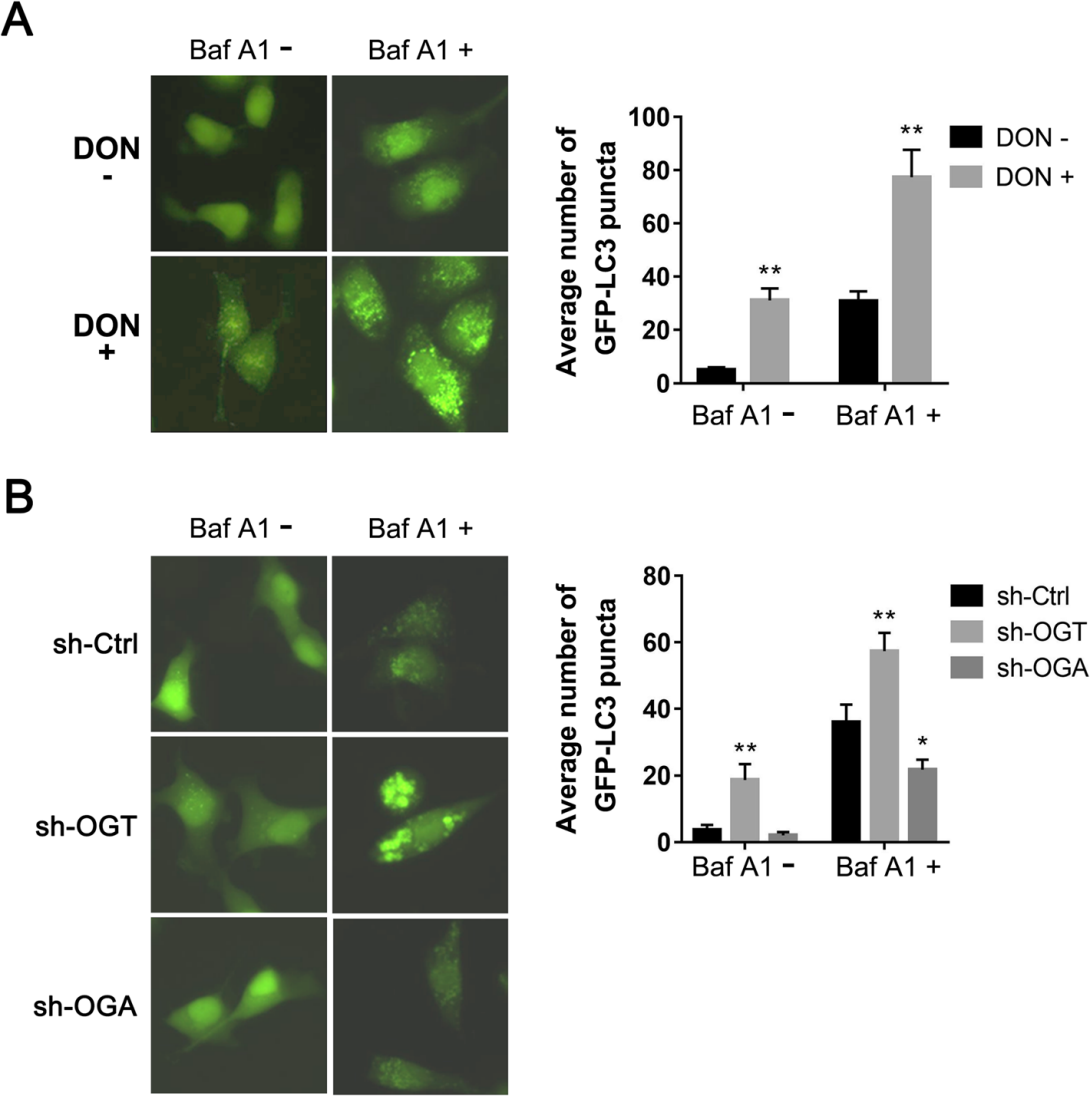
Blockage of O-GlcNAc promotes autophagy flux. **a –** The 5637-GFP-LC3 cells were pretreated with Baf A1 (10 nM) for 24 h. The cells were then treated with DON for another 16 h. GFP-LC3 fluorescence was captured with fluorescence microscopy after the treatment. **b –** Baf A1 was added to 5637-GFP-LC3 cells stably transfected with sh-OGT or sh-OGA. GFP-LC3 fluorescence was captured with fluorescence microscopy. The average number of GFP-LC3 puncta was calculated for 200 cells. Data in the histograms are shown as the means ± SD. *P < 0.05 and **P < 0.01

To define the origins of the increase in the number of GFP-LC3 puncta after O-GlcNAcylation inhibition, we pretreated 5637-GFP-LC3 cells with Baf A1, a compound that inhibits the fusion of the autophagosome and lysosome to repress autophagosome degradation. Baf A1 treatment dramatically increased the numbers of GFP-LC3 cytoplasmic puncta regardless of DON treatment (Fig. 3a). However, even in the presence of Baf A1, DON-mediated depletion of O-GlcNAcylation further enhanced the accumulation of autophagosomes. Similarly, Baf A1 promoted the enhancement of autophagosomes in OGT- or OGA-manipulated 5637-GFP-LC3 cells (Fig. 3b). Our results suggest that the increased number of GFP-LC3 cytoplasmic puncta after inhibition of O-GlcNAcylation are most probably due to escalated induction of autophagic flux, not the degradation blockage of autophagy vesicles.

### O-GlcNAcylation negatively regulates AMPK activity and ULK1-Ser555 phosphorylation in bladder cancer cells

**Fig. 4:**
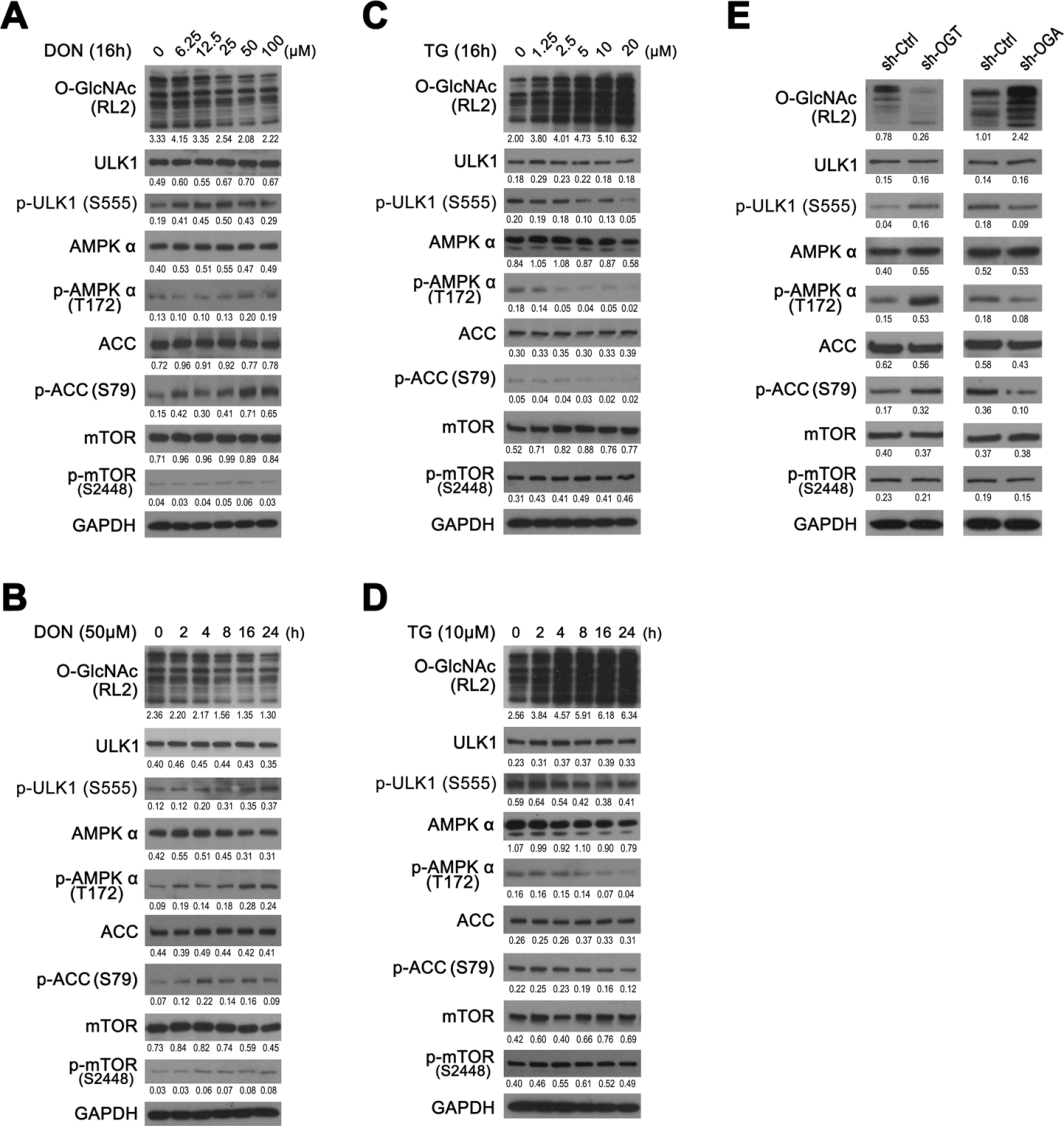
O-GlcNAc negatively regulates phosphorylation of AMPK and ULK1 in 5637 cells. **a through d** – Whole cell extracts were isolated from 5637 cells treated with DON or TG. Western blotting was used to determine the expression of the proteins. **a** and **b** – 5637-GFP-LC3 cells were treated with different doses of DON for 16 h (**a**) or with DON (50 μM) for different time points (**b**). **c** and **d** – The 5637-GFP-LC3 cells were treated with different doses of TG for 16 h (**c**) or with TG (10 μM) for different time durations (**d**). **e** – The expressions of proteins in 5637-GFP-LC3 cells with knockdown of OGT or OGA, and in the 5637 negative control

The ULK1 complex plays a central role in autophagy initiation by integrating signals from upstream sensors such as mTOR and AMPK [28, 29]. To explore the mechanism by which O-GlcNAcylation regulates the basal autophagy level in bladder cancer cells, we determined the phosphorylation of ULK1 and its upstream regulators, including AMPK and mTOR. As shown in Fig. 4a and b, 5637-GFP-LC3 cells treated with DON in incremental doses or time periods showed a general trend of decreased overall O-GlcNAcylation and gradually increased expression of p-ULK1 (S555), p-AMPKα (T172), and p-ACC (S79), which is a downstream effector of p-AMPK (Fig. 4a and b). However, the expression change of p-mTOR (S2448), a suppressor of ULK1, was uncertain when treated with DON. By contrast, when the cells were treated with TG, the overall level of O-GlcNAcylation was higher, and the expressions of p-ULK1(S555), p-AMPKα (T172), and p-ACC(S79) were downregulated (Fig. 4c and d). In addition, the expression of p-mTOR (S2448) in 5637 cells was not significantly changed after TG treatment.

To further validate the regulatory role of O-GlcNAcylation in AMPK–ULK1 activation, we determined phosphorylation of AMPK and ULK1 in OGT- or OGA-manipulated 5637 cells. Lower overall O-GlcNAcylation, upregulated expressions of p-ULK1, p-AMPKα and p-ACC, and unchanged expression of p-mTOR were found in 5637-sh-OGT cells (the left panel in Fig. 4e). By contrast, higher overall O-GlcNAcylation and downregulated expression of p-ULK1, p-AMPK and p-ACC were observed in 5637-sh-OGA cells, accompanied by unchanged expression of p-mTOR (the right panel in Fig. 4e). Opposite results were obtained as OGT or OGA was overexpressed in 5637 cells (Supplementary Fig. S1C). Next, we altered the levels of overall O-GlcNAcylation in RT4-GFP-LC3 cells by treatment with DON, TG, or shRNAs (sh-OGT or sh-OGA), in which similar results as in 5637 cells aforementioned were obtained (Supplementary Fig. S1D and E). These results indicate that the overall decrease in O-GlcNAcylation is associated with increased AMPK activity and ULK1-Ser555 phosphorylation in bladder cancer cells.

### AMPKα is required for O-GlcNAc-mediated regulation of autophagy

**Fig. 5:**
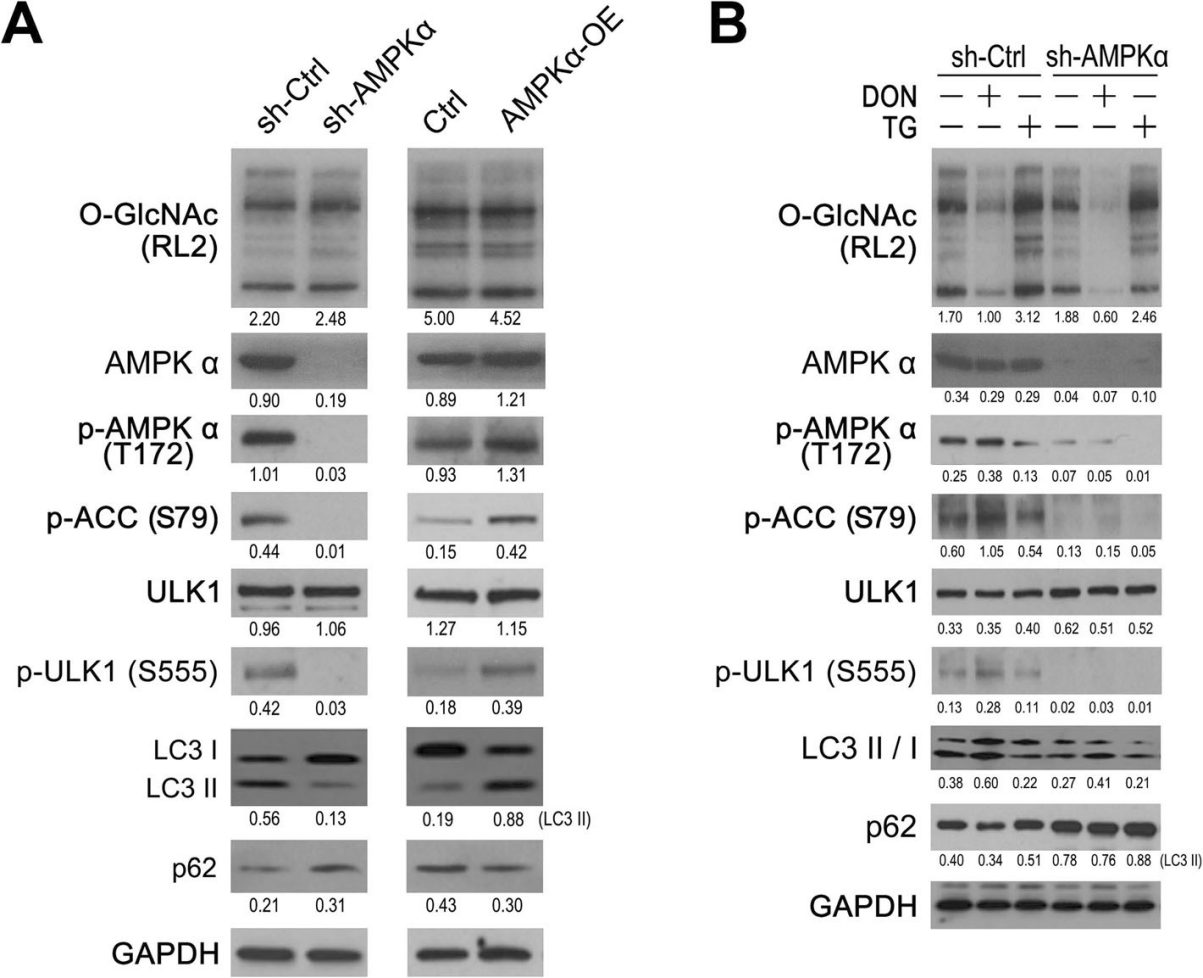
AMPK is required for O-GlcNAc-mediated regulation of autophagy. **a** – The expressions of O-GlcNAc (RL2), p-AMPKα (T172), p-ACC (S79), ULK1, p-ULK1(S555), LC3 and p62 in 5637 cells with AMPKα manipulations were measured using western blotting. **b** – 5637 cells with sh-AMPKα were treated with DON (50 μM) or TG (10 μM) for 16 h, and the expressions of the proteins listed above were measured using western blotting. GAPDH served as an internal control

ULK1 can be phosphorylated and activated by AMPK. Phosphorylation of ULK1 is crucial to autophagy initiation and progression. To verify the regulation of to autophagy by AMPK in bladder cancer cells, ULK1 phosphorylation and the levels of the autophagic markers (LC3 and p62) were determined in 5637 cells with AMPKα knockdown (sh-AMPKα) or overexpression (AMPKα-OE). ULK1-Ser555 phosphorylation was markedly suppressed in sh-AMPKα cells, but upregulated in AMPKα-OE cells as compared with the control cells with endogenous AMPKα (Ctrl; Fig. 5a). Autophagy was respectively inhibited or enhanced by AMPKα knockdown or overexpression, based on the expression changes of LC3 II and p62. However, overall O-GlcNAcylation remained at a constant level as AMPKα expression was altered.

Next, to determine whether the O-GlcNAc-mediated regulation of autophagy depends on AMPKα, DON or TG were added to the sh-AMPKα and control cells. Treatment of 5637 negative control cells with DON (Fig. 5b, left) significantly suppressed overall O-GlcNAcylation, increased the expressions of p-AMPKα, p-ACC and p-ULK1, and enhanced autophagy, as seen in the increased LC3 II and decreased p62 expression. TG treatment had the opposite effect. Importantly, knockdown of AMPKα diminished the effects of DON and TG on the expression of p-ULK1 and autophagy in 5637 cells (Fig. 5b, right). These data suggest that AMPKα is required for the O-GlcNAc-mediated regulation of autophagy in bladder cancer cells.

### AMPK is directly O-GlcNAcylated in bladder cancer cells

**Fig. 6:**
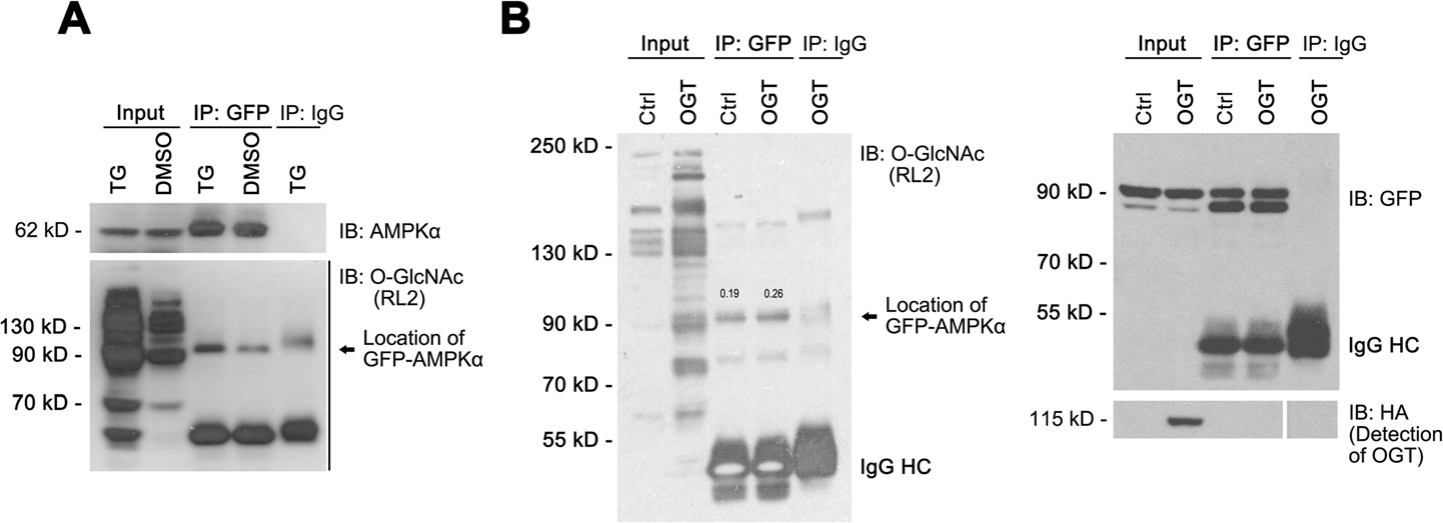
AMPK is O-GlcNAcylated. Co-IP was used to test the relationship between O-GlcNAc and AMPK. **a** – The 5637-GFP-AMPKα cells were treated with TG and cell extracts were used for the Co-IP with an anti-GFP antibody. IP products were subjected to detect the presence of O-GlcNAc (using western blotting with RL2 antibody) and that of AMPKα fused with GFP (using AMPKα antibody). **b** – The 5637 cells were transfected with an empty vector (Ctrl) or OGT-overexpression (OGT-OE) construct, and then the extracts were used for the Co-IP with an anti-GFP antibody and further analyzed using western blotting with RL2 antibody (left panel) or GFP and HA antibody (right panel)

Considering AMPKα is required for O-GlcNAc-mediated regulation of autophagy, it is necessary to examine whether AMPK is O-GlcNAcylated directly. AMPKα-GFP fusion protein was immunoprecipitated with a GFP antibody from extracts of 5637 cells transfected with GFP-AMPKα (5637-GFP-AMPKα) and detected using AMPKα and O-GlcNAc antibodies (Fig. 6a). Immunoblots showed that the O-GlcNAcylation level of AMPKα markedly increased in cells treated with TG relative to the control (DMSO). In addition, the AMPKα-GFP fusion protein immunoprecipitated with the GFP antibody was detected using an O-GlcNAc antibody (Fig. 6b, left panel), and a GFP antibody as an internal control (Fig. 6b, right panel) in 5637-GFP-AMPKα cells with overexpression of OGT. Immunoblots showed that the O-GlcNAcylation level of AMPKα increased after OGT overexpression in 5637 cells (Fig. 6b, left panel). Thus, AMPKα can be O-GlcNAcylated, suggesting that the direct O-GlcNAcylation of AMPKα is responsible for the O-GlcNAc-mediated regulation of autophagy in bladder cancer cells.

## Discussion

Nutrient-sensitive O-GlcNAc modification regulates proteins in diverse cellular signaling pathways in mammalian cells [30]. Increased total O-GlcNAcylation is detected in cancer cells derived from breast, prostate, colon, lung, pancreas and bladder tumors [5], suggesting that the process has an oncogenic role.

Autophagy is a cellular pathway responsible for protein and organelle degradation. It is induced by various stresses, including nutrient, oxygen and growth factor deprivation and chemotherapeutics [18, 19]. Although studies have confirmed that O-GlcNAcylation or OGT has a role in the regulation of autophagic flux in neurodegenerative diseases [31, 32], there is no evidence to show this regulation in cancer. In this study, we demonstrated that autophagy is negatively regulated by O-GlcNAcylation in bladder cancer cells.

ULK1 integrates signals from upstream sensors, such as mTOR and AMPK, to initiate autophagy. We observed the activation of ULK1 and its upstream kinase AMPK in O-GlcNAc depletion in bladder cancer cells. Previous research reported that AMPK activation is responsible for reduced phosphorylation of mTORC1 at S2448, and that this is coordinated with decreased mTORC1 activity (represented by S6 S235/236 phosphorylation) [25]. Similarly, Rosner et al. showed that S2448-phosphorylated mTOR binds to both the mTORC1 component raptor and the mTORC2 component rictor [33]. Using chemical inhibitors of the mTOR kinase and of PI3K, it was found that downregulation of mTOR S2448 phosphorylation correlates with decreased mTORC1 activity but can occur decoupled from the effects on mTORC2 activity (represented by the phosphorylation of Akt S473). Therefore, we evaluated mTORC1 activity via detection of phosphorylation of mTOR S2448 under the O-GlcNAc treatments. We found that p-mTORC1 S2448 was not strikingly affected by manipulations of O-GlcNAc. Thus, mTORC1 may not be a major target mediating the induction of autophagy in O-GlcNAc depletion.

Here, we found that blockage of O-GlcNAc induces cell autophagy in bladder cancer cells through an mTOR-independent pathway. O-GlcNAcylation of AMPK suppressed the activity of AMPK, which inhibited the activity of ULK1 and resulted in cell autophagy.

Ferrer et al. previously reported that AMPK-activity increases when O-GlcNAcylation of proteins is reduced, as measured by the phosphorylation of raptor through AMPK, which is consistent with the idea that mTORC1-activity is reduced by activation of AMPK. However, it was undetermined whether O-GlcNAcylation of AMPK (and hence activity of AMPK) were promoted under the analyzed conditions. It was shown that a high cellular energy level (methyl pyruvate) and OGT knockdown or decreased O-GlcNacylation can no longer activate AMPK [23].

Previous studies have shown that AMPKα and γ subunits are O-GlcNAcylated directly in skeletal muscle cells [34]. AMPK can also alter the O-GlcNAcylation of other proteins to regulate numerous nutrient-sensitive processes for life. Here, we found that AMPKα was O-GlcNAcylated under basal conditions. The activity of p-ULK1 was blocked as AMPK is O-GlcNAcylated. Luo B et al. previously reported that increased AMPK activity is associated with enhanced O-GlcNAcylation of AMPK in adipocytes, raising the possibility that AMPK-signaling is regulated differently by O-GlcNAcylation in various cell types. More effort is needed to identify the potential O-GlcNAcylation site(s) of AMPK and characterize the resultant functions of the modification in different cell types.

## Conclusions

We found that O-GlcNAcylation negatively regulates autophagic flux by targeting the AMPK-ULK1 pathway in bladder cancer cell lines. These findings might provide a rationale for exploring the role of O-GlcNAcylation in cancer development and progression.

## Supplementary information

**Additional file 1 Supplementary Fig. S1. A.** Autophagic flux was observed in RT4-GFP-LC3 cells treated with DON and TG. RT4-GFP-LC3 cells untreated (Mock) and treated by DON (50 μM) and TG (10 μM) for 16 h were subjected to detection of the GFP-LC3 fluorescence with fluorescence microscopy. The average number of GFP-LC3 puncta was calculated in 200 cells (lower panel). **B.** Autophagic flux was observed in RT4-GFP-LC3 cells with stable downregulated expression of OGT or OGA. OGT or OGA was stably silenced with shRNAs in RT4-GFP-LC3 cells, and then GFP-LC3 fluorescence was captured with fluorescence microscopy in the cells. The average number of GFP-LC3 puncta was calculated in 200 cells (lower panel). **C.** Protein expression in 5637 cells with overexpression of OGT or OGA. OGA or OGT was overexpressed in 5637-GFP-LC3 cells. Proteins were extracted from cells and determined by western blot assay. GAPDH was served as an internal control. **D** and **E.** Protein expression in RT4 cells with altered levels of O-GlcNAcylation. (D) RT4-GFP-LC3 cells untreated (Mock) and treated by DON (50 μM) and TG (10 μM) for 16 h were subjected to detection of protein expression with western blot assay. (E) OGT or OGA was stably silenced with shRNAs in RT4-GFP-LC3 cells. Protein expression levels in the cells (sh-OGT and sh-OGA) and negative control cells (sh-Ctrl) were determined with western blot assay. GAPDH was served as an internal control.

## Data Availability

The datasets used and/or analyzed during this study are available from the corresponding author on reasonable request.
