# Supplementary figures and images for: Blockage of O-linked GlcNAcylation induces AMPK-dependent autophagy in bladder cancer cells
Source: Cell Mol Biol Lett. 2020 Mar 10;25:17. doi: 10.1186/s11658-020-00208-x (PMC7063793; doi:10.1186/s11658-020-00208-x)

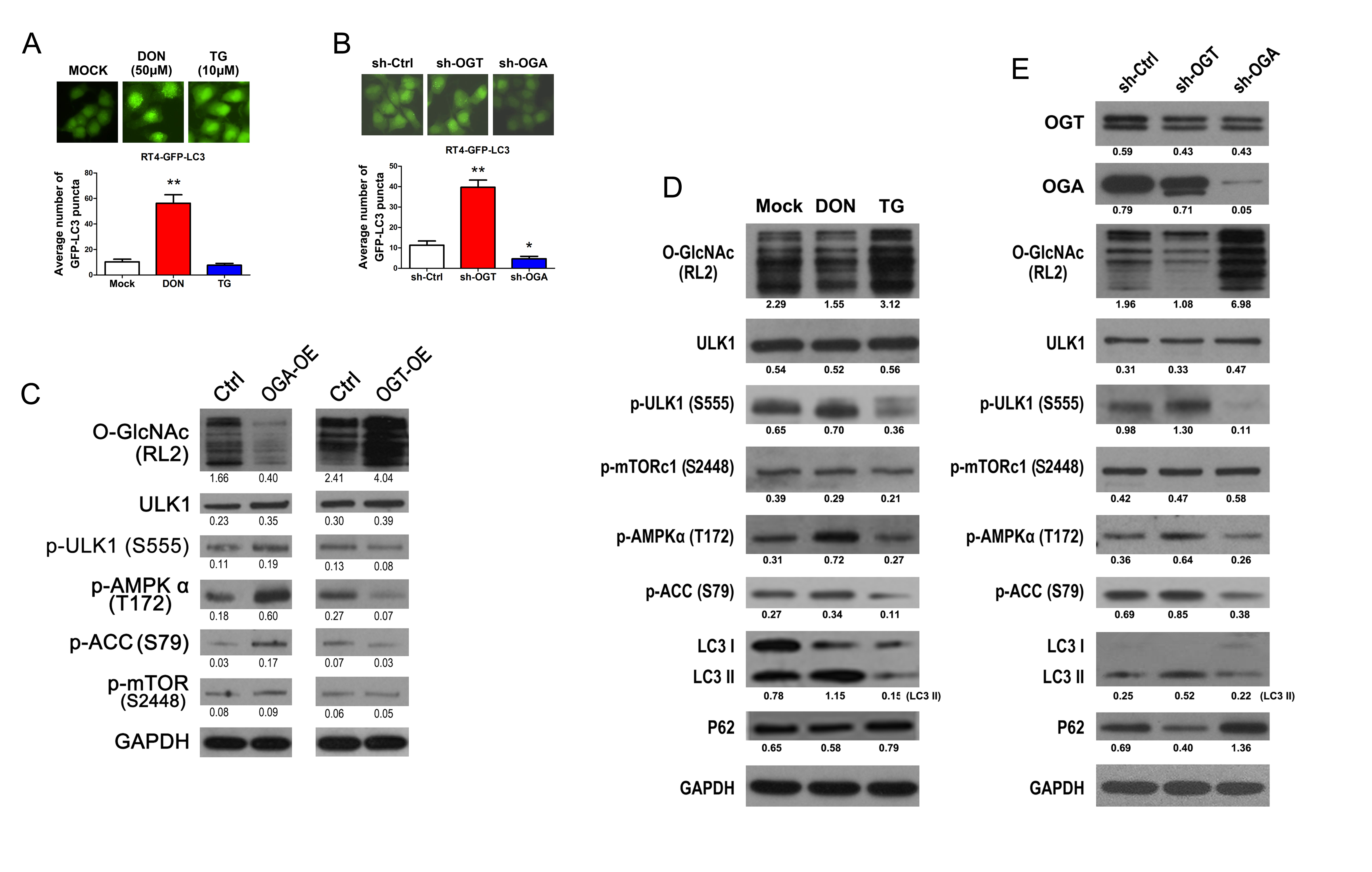

Supplement: Supplementary file 1 — Additional file 1 Supplementary Fig. S1. A. Autophagic flux was observed in RT4-GFP-LC3 cells treated with DON and TG. RT4-GFP-LC3 cells untreated (Mock) and treated by DON (50 μM) and TG (10 μM) for 16 h were subjected to detection of the GFP-LC3 fluorescence with fluorescence microscopy. The average number of GFP-LC3 puncta was calculated in 200 cells (lower panel). B. Autophagic flux was observed in RT4-GFP-LC3 cells with stable downregulated expression of OGT or OGA. OGT or OGA was stably silenced with shRNAs in RT4-GFP-LC3 cells, and then GFP-LC3 fluorescence was captured with fluorescence microscopy in the cells. The average number of GFP-LC3 puncta was calculated in 200 cells (lower panel). C. Protein expression in 5637 cells with overexpression of OGT or OGA. OGA or OGT was overexpressed in 5637-GFP-LC3 cells. Proteins were extracted from cells and determined by western blot assay. GAPDH was served as an internal control. D and E. Protein expression in RT4 cells with altered levels of O-GlcNAcylation. (D) RT4-GFP-LC3 cells untreated (Mock) and treated by DON (50 μM) and TG (10 μM) for 16 h were subjected to detection of protein expression with western blot assay. (E) OGT or OGA was stably silenced with shRNAs in RT4-GFP-LC3 cells. Protein expression levels in the cells (sh-OGT and sh-OGA) and negative control cells (sh-Ctrl) were determined with western blot assay. GAPDH was served as an internal control. [file 11658_2020_208_MOESM1_ESM.tif]
